# Combination of whole body cryotherapy with static stretching exercises reduces fatigue and improves functioning of the autonomic nervous system in Chronic Fatigue Syndrome

**DOI:** 10.1186/s12967-022-03460-1

**Published:** 2022-06-17

**Authors:** Sławomir Kujawski, Joanna Słomko, Beata R. Godlewska, Agnieszka Cudnoch-Jędrzejewska, Modra Murovska, Julia L. Newton, Łukasz Sokołowski, Paweł Zalewski

**Affiliations:** 1 Department of Exercise Physiology and Functional Anatomy, Ludwik Rydygier Collegium Medicum in Bydgoszcz Nicolaus Copernicus University in Toruń, Świętojańska 20, 85-077 Bydgoszcz, Poland; 2 Department of Psychiatry, University of Oxford, Oxford, OX3 7JX UK; 3 Department of Experimental and Clinical Physiology, Laboratory of Centre for Preclinical Research, Warsaw Medical University, 1b Banacha Street, 02-097 Warsaw, Poland; 4 Institute of Microbiology and Virology, Riga Stradinš University, Riga, 1067 Latvia; 5 Population Health Sciences Institute, The Medical School, Newcastle University, Framlington Place, Newcastle-upon-Tyne, NE2 4HH UK

**Keywords:** Myalgic encephalomyelitis, Chronic fatigue syndrome, Autonomic nervous system, Cold therapy, Brain fog

## Abstract

**Background:**

The aim of this study was to explore the tolerability and effect of static stretching (SS) and whole body cryotherapy (WBC) upon fatigue, daytime sleepiness, cognitive functioning and objective and subjective autonomic nervous system functioning in those with Chronic Fatigue Syndrome (CFS) compared to a control population.

**Methods:**

Thirty-two CFS and eighteen healthy controls (HC) participated in 2 weeks of a SS + WBC programme. This programme was composed of five sessions per week, 10 sessions in total.

**Results:**

A significant decrease in fatigue was noted in the CFS group in response to SS + WBC. Some domains of cognitive functioning (speed of processing visual information and set-shifting) also improved in response to SS + WBC in both CFS and HC groups. Our study has confirmed that WBC is well tolerated by those with CFS and leads to symptomatic improvements associated with changes in cardiovascular and autonomic function.

**Conclusions:**

Given the preliminary data showing the beneficial effect of cryotherapy, its relative ease of application, good tolerability, and proven safety, therapy with cold exposure appears to be an approach worth attention. Further studies of cryotherapy as a potential treatment in CFS is important in the light of the lack of effective therapeutic options for these common and often disabling symptoms.

**Supplementary Information:**

The online version contains supplementary material available at 10.1186/s12967-022-03460-1.

## Background

Chronic Fatigue Syndrome (CFS) is a condition that could significantly reduce the opportunity to undertake normal daily activities, in more severe forms might lead to inability to undertake even part time work and being chronically bedridden. There are no specified diagnostic markers nor differing diagnostic criteria commonly established [1].

The global prevalence of CFS ranges between 0.4% and 2.5% and is growing; CFS affects 0.3–3.3% of the US population, 2.6% in the UK and 3% in China. In Poland, in cohort of people suffering from chronic fatigue the prevalence of CFS is 5% from and is associated with a significant symptom burden and impaired quality of life [2].Autonomic nervous system (ANS) dysfunction is one of the hallmark parts of mechanism underlying pathology of CFS [3]. Heart rate variability (HRV) could serve as a biomarker on which basis healthy and diseased states distinguished [4] and it seems to be a useful biomarker of stress response and adaptation [5].

The Institute of Medicine (IOM) report in 2015 underlined neurocognitive impairment as one of key symptoms in many people with ME/CFS [6]. Cognitive function impairment might comprise of decreased performance in short-term memory, attention, processing function, what in turn might lead to disturbance of everyday activities including work, leisure activities, reducing perceived quality of life [6]. 50% to 85% of ME/CFS patients note subjective cognitive impairments [7]. A set of symptoms including, inter alia, general slowdown in response speed to tasks that require simple and complex information processing as well as the ability to focus on a task was is referred commonly to as a “brain fog” [8]. Cognitive disturbances seen in ME/CFS patients might be restricted to a decrease in basic processing speed and not related to depression severity [8, 9]. Verbal memory, visuo-spatial memory and linguistic fluency in CFS patients appear to be similar to healthy controls [10]. Nevertheless, effortful tasks based on planned and self-ordered responses which requires the use of short-term memory capacity seems to be disturbed because of reduced attention capacity [11]. A recent revision of guidelines for CFS patients has stated that there are currently no approved treatments for CFS [12].

Voluntary exposure on extremely low temperatures (e.g. local and whole-body cryotherapy (WBC) is one of thermotherapeutic stimuli that is frequently used in Eastern Europe as a form of physical therapy. WBC is comprised of a short time (2–3 min) exposure in ambient temperature from − 110 to − 160 °C to stimulate the body by the intense peripheral hypothermia of virtually its entire area and such controlled exposure is considered to be safe [13, 14]. Repeated WBC sessions are mostly used in form of therapeutic programme with many potential application: to decrease pain, chronic inflammation, as an additional therapy in acute injures soft tissue, rheumatic disease and neurodegenerative conditions and in process of recovery in athletes [14, 15].

WBC activates physiological mechanisms which goal is to maintain a constant body core temperature. It might include rapid and short-term regulatory mechanisms predominantly focused upon cardiovascular and autonomic nervous system functions [16–18]. Peripheral blood vessels constriction might lead to a reduction in temperature of the cold-exposed skin area. In consequence, it might decrease the perfusion of the skin vascular bed reducing convective and conductive heat loss [19, 20].

In Our previous study on effects of static stretching (SS) and WBC we have applied network analysis to assess differences in the allostatic response to intervention in CFS in comparison to healthy controls [21]. Such analysis was helpful in relieving the interaction between physiological systems in response to stressor in form of applied intervention. One of cardinal symptoms of CFS is post-exertional malaise (PEM) which could be induced by physical and/or cognitive activity [6]. Therefore, in the previous study [21] we have examined if WBC + SS is included in one of group of stressors which might trigger PEM, which in turn would lead to a significant disturbance of homeostasis and increase in symptoms severity in CFS patients. In the above study we would like to analyze the effects of WBC + SS on each parameter separately, to examine if its effective in reducing fatigue and severity of other symptoms related to CFS.

The aim of current study was to explore the tolerability and effect of static stretching and WBC upon fatigue, daytime sleepiness, cognitive functioning and objective and subjective autonomic nervous system functioning in those with CFS compared to a control population.

## Results

### Whole study group analysis

**Fig. 1:**
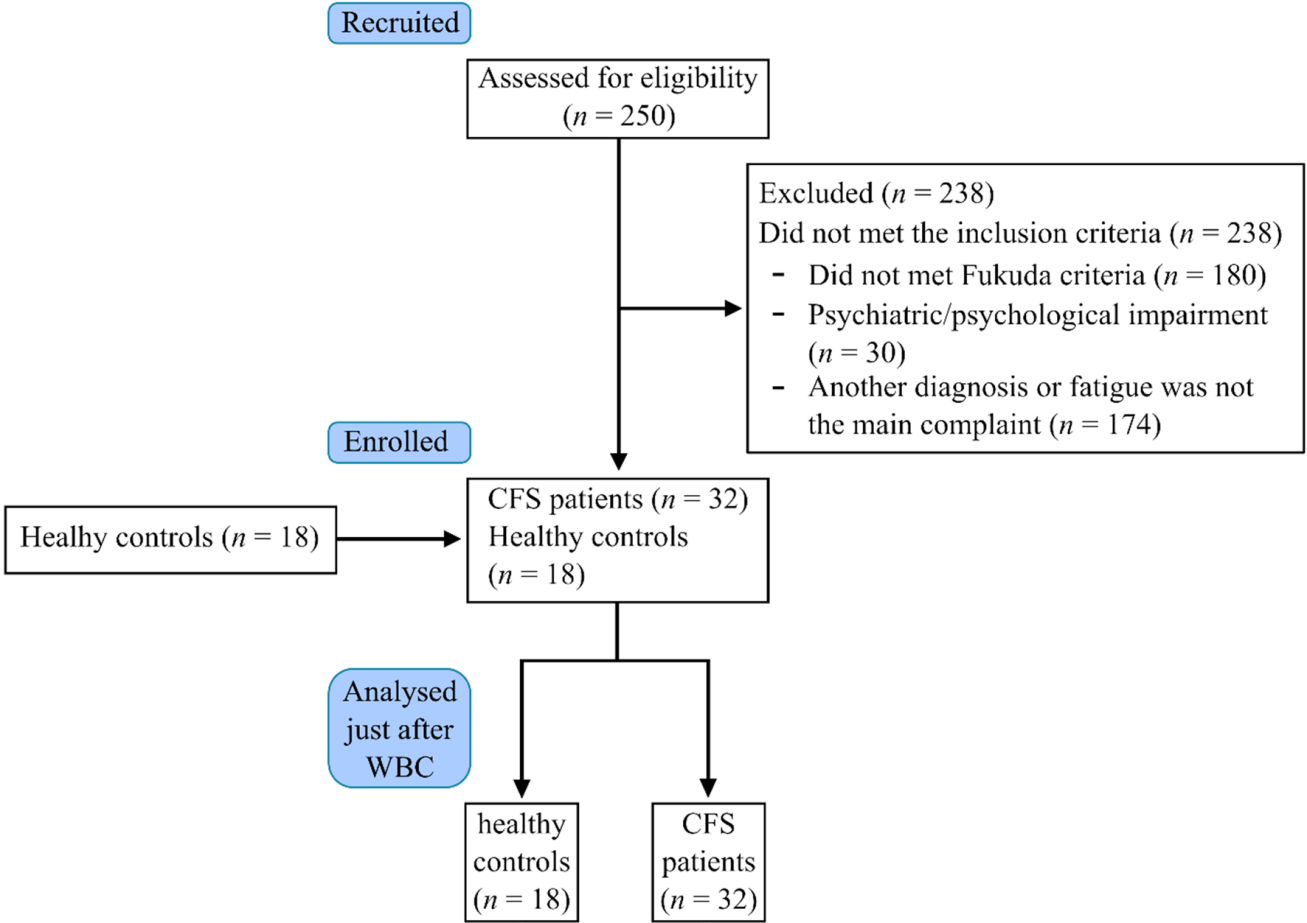
Flow chart of the study

After 250 patients were initially assessed for eligibility, 238 patients were excluded. The study included 32 CFS patients and 18 healthy controls (HC), no participants were lost to follow-up (Fig. 1). Baseline characteristics were similar between the two groups. As shown in Additional file 1: Table S1 no differences were found in age, body composition or BMI between the groups.

The mean ± standard deviation (SD) of age for CFS patients, and HC were 36.7 ± 8.4, and 38.4 ± 7.9 respectively. Mean length of history for the CFS group was 3.6 ± 2.9 years (range 1–12 years). The majority (53.1%) of those with CFS had symptoms for 6 months to 2 years with 31.2% having symptoms for 3–5 years, while symptoms between 6 and 10 years (12.5%) and more than 10 years were the least prevalent (3.1%). The most common comorbidities in patients with CFS were vertigo (40.6%) followed by fibromyalgia (31.2%), cognitive impairment, migraine and arthritis (28.1%). (Additional file 1: Table S2). The vast majority of those with CFS described fatigue (87.5%), impaired concentration and difficulty retrieving words (87.5%), muscle pain (87.5%), memory disturbances (84.4%). The majority of CFS and HC were from a specialist professional group (engineers). Additional file 1: Table S3 shows detailed information about the total CFS group. (Additional file 1: Table S3).

### *Effects of WBC* + *SS on self-reported measures of fatigue and daytime sleepiness in the CFS group*

**Fig. 2:**
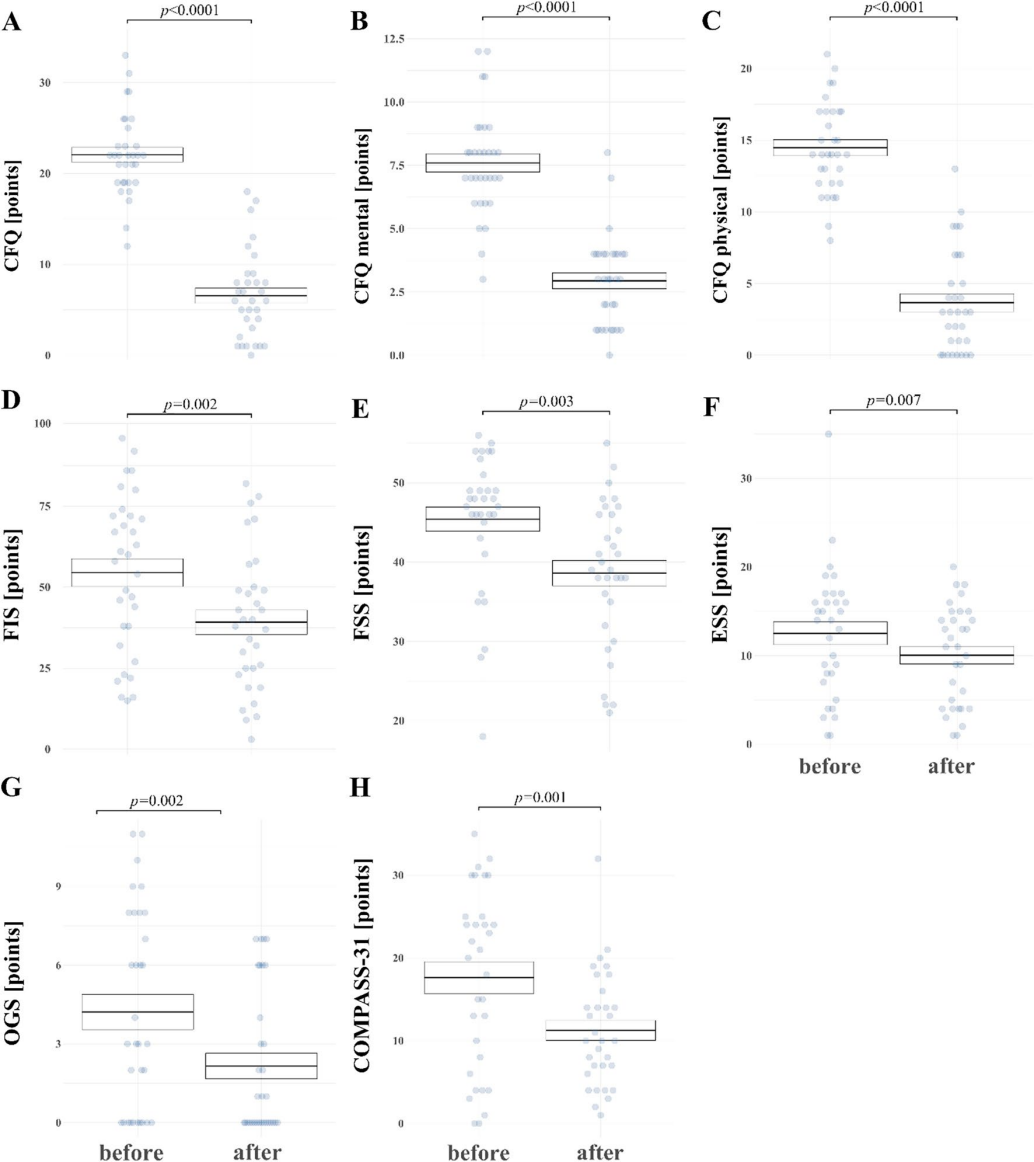
CFS and HC mean values (± SE) for points in scales: **A** CFQ-Chalder Fatigue Questionnaire, **B** CFQ-Chalder Fatigue Questionnaire mental domain, **C** CFQ-Chalder Fatigue Questionnaire physical domain, **D** FIS-Fatigue Impact Scale, **E** FSS-Fatigue Severity Scale, **F** ESS-Epworth Sleepiness Scale, **G** OGS-Orthostatic Grading Scale, **H** Compass-COMPASS-31 scale. *P*-values reported were adjusted using Holm correction

Figure 2 presents effects of WBC + SS in CFS group only. After WBC + SS, the CFS group showed significant improvement in self-reported fatigue (CFQ total; CFQ mental and physical domain; FIS, FSS), orthostatic intolerance (OGS), daily sleepiness (ESS), and subjective autonomic symptoms (COMPASS 31, OGS) and daytime sleepiness (ESS), respectively. CFQ score decreased from 22.1 ± 4.5 before WBC + SS to 6.6 ± 4.8 after (F = 176.1, *p* < 0.0001) (Fig. 2A). CFQ physical and mental fatigue decreased significantly (F = 166.4, *p* < 0.0001 and F = 92.9, *p* < 0.0001, respectively) (Fig. 2B, C). FIS decreased from 54.5 ± 24.2 before WBC + SS to 39.2 ± 21.3 after WBC + SS (F = 12.8, *p* = 0.002) (Fig. 2D), FSS decreased from 45.4 ± 8.7 before WBC + SS to 38.6 ± 9.1 after (F = 10.2, *p* = 0.003) (Fig. 2E). ESS score decreased from 12.5 ± 7.3 before WVC to 10.1 ± 5.6 (*p* = 0.007) (Fig. 2F). OGS score decreased from 4.2 ± 3.8 before to 2.2 ± 2.8 after (F = 13.6, *p* = 0.002) (Fig. 2G), COMPASS 31 score decreased from 17.6 ± 10.8 before WBC + SS to 11.3 ± 6.8 (F = 14.7, *p* = 0.001) (Fig. 2H).

### Cardiac and autonomic assessment before,- and after WBC + SS intervention: comparison between the CFS and HC groups

**Fig. 3:**
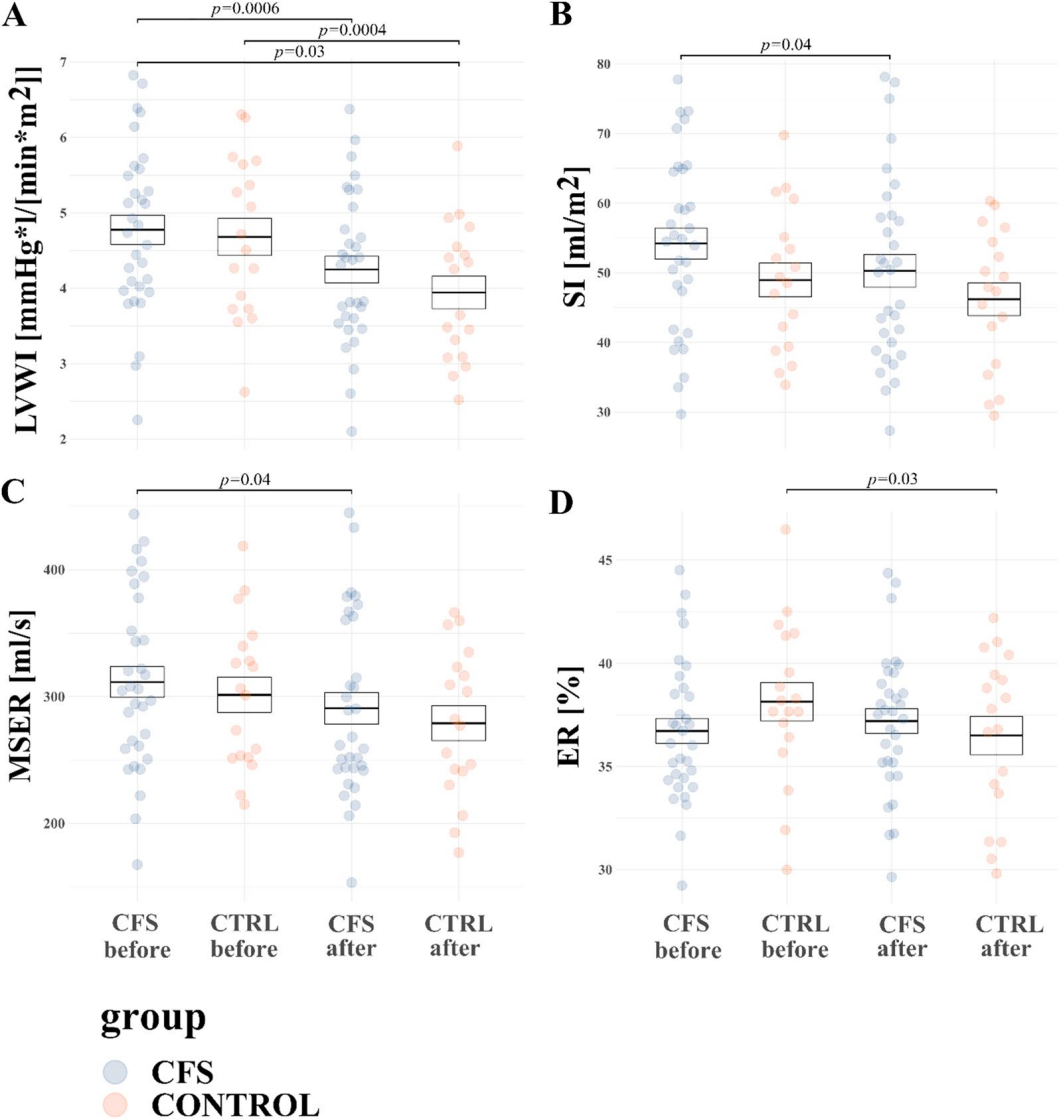
CFS and HC mean values (± SE) at rest of **A** LVWI (left ventricular work index); **B** SI (stroke index); **C** MSER (Mean Systolic Ejection Rate); **D** ER (Ejection Rate). *P*-values from ANOVA conducted on linear mixed models are reported for the effects of group (HC vs CFS), WBC + SS (before vs after) and interaction between those factors

HR was changed by interaction of effects of WBC + SS and group (F = 9.1, *p* = 0.004). In CFS group HR tended to increase, whilst in the control group HR tended to decrease after WBC + SS, however, the results of the post-hoc tests were not significant (*p* > 0.05). WBC + SS changed BP in both groups: both sBP, dBP and mBP tended to decrease in both groups in response WBC + SS, however, again the results of post-hoc tests were not significant (*p* > 0.05). WBC + SS changed IC in both groups (*p* = 0.01). Contractility index (IC) tended to decrease in both groups in response WBC + SS, however, results of post-hoc tests were not significant (*p* > 0.05). Left ventricular work index (LVWI) decreased in both groups in response WBC + SS (F = 36.2, *p* < 0.0001). In CFS group LVWI decreased from 4.8 ± 0.2 mmHg*l/[min*m^2^] to 4.2 ± 0.2 after WBC + SS (p = 0.0006), while in control group LWVI decreased from 4.7 ± 0.3 mmHg*l/[min*m^2^] to 3.9 ± 0.2, (p = 0.0004) (Fig. 3A). Moreover, change in SI in response to WBC + SS was noted (F = 8.6, *p* = 0.005). Post-hoc test indicates significant decrease of SI in response to WBC + SS in CFS group (54.2 ± 2.1 ml/m^2^ before vs 50.3 ± 2.2 after WBC + SS, *p* = 0.04) (Fig. 3B). Significant interaction between effects of group and WBC + SS were noted in the case of total fluid content (TFC) (F = 8.2, *p* = 0.006). In CFS group TFC tended to increase, while in control group TFC tended to decrease after WBC + SS however, results of post-hoc tests were not significant (*p* > 0.05). Significant interaction between effects of group and WBC + SS on mean systolic ejection rate (MSER) was noted (F = 12.8, *p* = 0.0008). In both groups MSER tended do decrease, post-hoc test revealed significant decrease in CFS group 311.6 ± 69 ml/s before WBC + SS vs 290.9 ± 70.6 after WBC + SS, *p* = 0.04) (Fig. 3C). Significant interaction between effects of group and WBC + SS on ejection rate (ER) was noted (F = 9.5, *p* = 0.003). In CFS group ER tended to increase, while in control group ER tended to decrease after WBC + SS, post-hoc tests showed that ER decreased in control group (38.2 ± 3.9% before WBC + SS vs 36.5 ± 3.9 after WBC + SS, *p* = 0.03) (Fig. 3D). Additional file 1: Table S4 presents data on resting values of cardiovascular function indicators for subjects with CFS and HC.

**Fig. 4:**
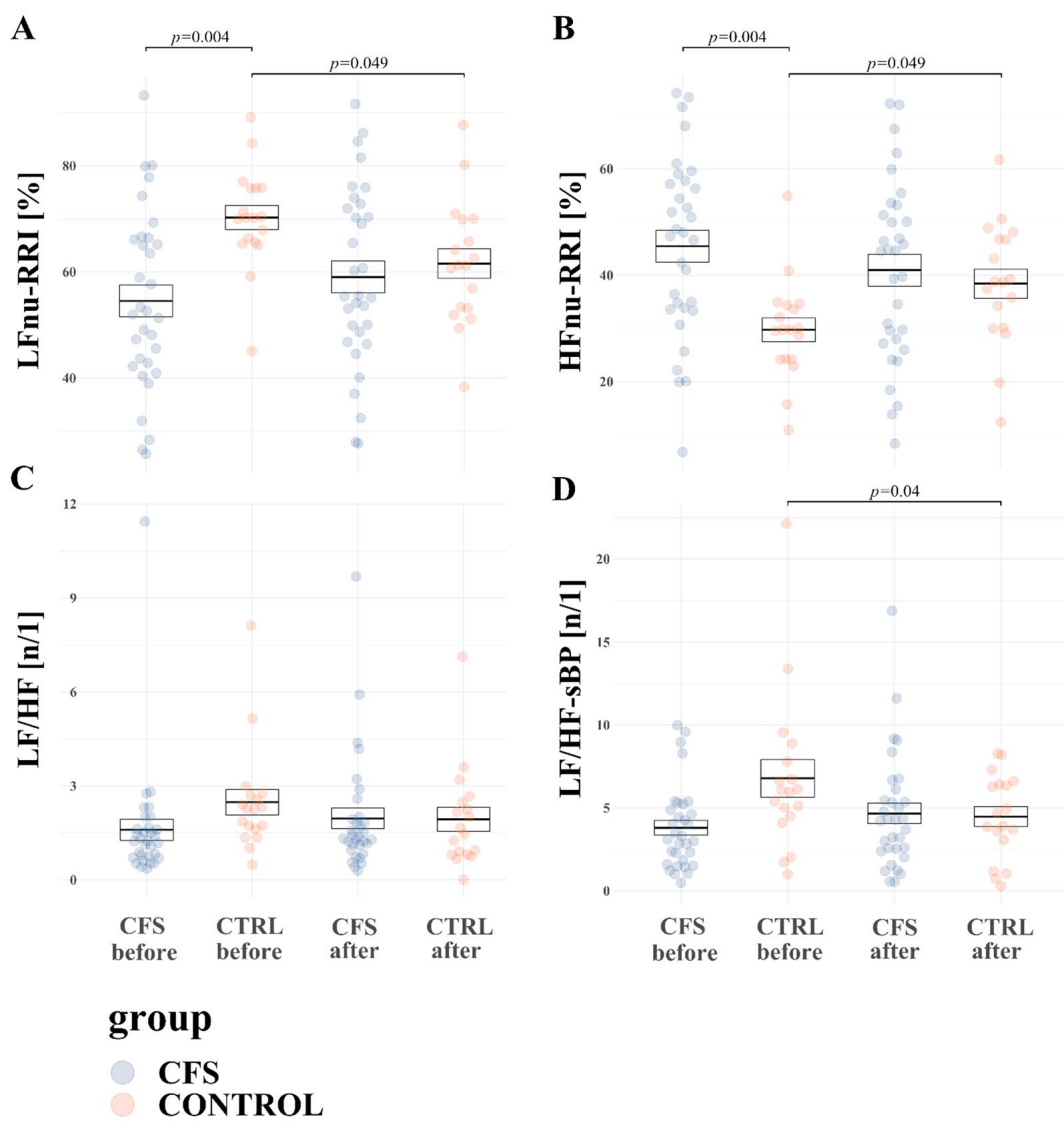
CFS and HC mean values (± SE) at rest of **A** LFnu RRI (normalized units of low-frequency RRI); **B** HFnu RRI (normalized units of high-frequency RRI); **C** LF/HF (ratio of LFnu-dBP to HFnu-RRI); **D** LF/HF sBP (ratio of LFnu sBP fo HFnu sBP)

After the WBC + SS programme, there was significant interaction between the effect of WBC + SS and group for LFnu-RRI (F = 10.7, *p* = 0.002), which decreased in HC group (from 70.3 ± 3.5 before WBC + SS vs 61.6 ± 3.6 after WBC + SS, *p* = 0.049), and increased in the CFS group without statistical significance (p > 0.05). LFnu-RRI was higher in the control group vs CFS patients before WBC + SS (54.5 ± 2.6 in CFS vs 70.3 ± 3.5 in control group before WBC + SS, *p* = 0.004) (Fig. 4A). In addition, a significant interaction between the effect of WBC + SS and group was noted for HFnu-RRI (F = 10.7, *p* = 0.002), which increased in HC group (from 70.3 ± 3.5 before WBC + SS vs 61.6 ± 3.6 after WBC + SS, *p* = 0.049), and decreased in the CFS group without statistical significance (*p* > 0.05). HFnu-RRI was lower in control group vs CFS patients before WBC + SS (45.5 ± 2.6 in CFS vs 29.7 ± 3.5 in the control group before WBC + SS, *p* = 0.004) (Fig. 4B). LF/HF changes are shown of Fig. 4C. Both LF/HF-RRI and LF/HF values were affected by interaction of the effects of WBC + SS and group (F = 5, *p* = 0.03 and F = 4.7, *p* = 0.03): both values tended to decrease in control and increase in CFS group, however changes were not statistically significant (*p* > 0.05). LF/HF dBP changed by interaction of effects of WBC + SS and group (F = 9, *p* = 0.004): it tended to decrease in control group and increase in CFS, however post-hoc analysis revealed no significant results. HFnu-sBP changed by interaction of effects of WBC + SS and group (F = 4.2, *p* = 0.047): it tended to increase in the control group and decrease in CFS, however post-hoc analysis revealed no significant results. LF/HF sBP changed by interaction of effects of WBC + SS and group (F = 9.5, *p* = 0.003): it tended to decrease in control group and increase in CFS. Post-hoc analysis revealed a significant decrease of LF/HF sBP in control group in response to WBC + SS (6.8 ± 0.8 before WBC + SS vs 4.5 ± 0.8 after, *p* = 0.04) (Fig. 4D). Data on effects of WBC + SS on resting values of autonomic measures in CFS and HC groups is summarized in Additional file 1: Table S5.

### Cognitive function assessment before,- and after WBC + SS intervention: comparison between the CFS and HC groups

**Fig. 5:**
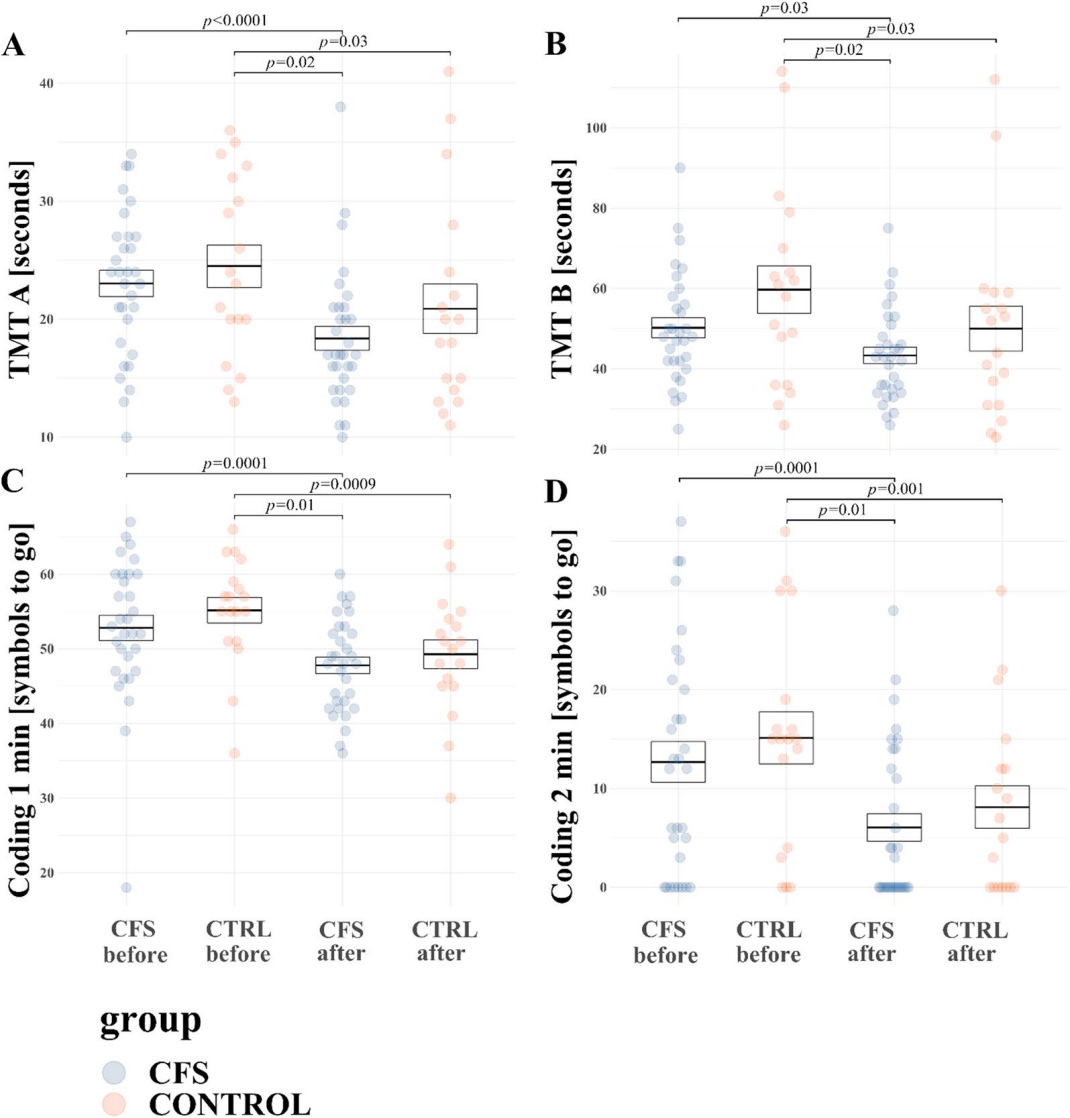
CFS and HC mean values (± SE) at rest of **A** TMT A (Trial Making Test part A); **B** TMT B (Trial Making Test part B); **C** Coding 1 min (number of symbols left to go after 1 min in Coding); **D** Coding 2 min (number of symbols left to go after 2 min in Coding)

A significant effect of WBC + SS was noted in time of execution of TMT A and TMT B, and digits left after the 1st and 2nd minute in Coding (Additional file 1: Table S6). WBC + SS was associated with decrease (i.e. improvement) in time of execution of TMT A (F = 29.5, *p* = 0.000002). Post-hoc analysis revealed that TMT A result decreased in the CFS group (from 23 ± 6.2 s before WBC + SS to 18.4 ± 5.7 after WBC + SS, *p* < 0.0001) and the control group (from 24.5 ± 7.6 s before vs 20.9 ± 8.9 after WBC + SS, *p* = 0.03). Moreover, the result of TMT A was significantly lower (better) after WBC + SS in the CFS group compared to the control group before WBC + SS (18.4 ± 5.7 s vs 24.5 ± 7.6, respectively, *p* = 0.02) (Fig. 5A). In addition, WBC + SS was associated with a decrease (i.e. improvement) in time of execution of TMT B (F = 16.6, *p* = 0.005). Post-hoc analysis revealed that TMT B result decreased in the CFS group (from 50.2 ± 13.9 s before WBC + SS to 43.3 ± 11.3 after WBC + SS, *p* = 0.03) and the control group (from 59.7 ± 25 s before vs 50 ± 23.7 after WBC + SS, *p* = 0.03). Moreover, the result of TMT B was significantly lower (better) after WBC + SS in the CFS group compared to the control group before WBC + SS (43.3 ± 11.3 s vs 59.7 ± 25, respectively, *p* = 0.02) (Fig. 5B). No significant effects on difference between the result of TMT B and A was noted *(p* > 0.05). WBC + SS led to reduction (i.e. improvement) in the number of digits left in coding after 1st and 2nd minute (F = 37.4, *p* = 0.0000002, F = 37.6, *p* = 0.0000001, respectively). Post-hoc analysis showed that in CFS group number of symbols left after 1st minute decreased from 52.8 ± 9.5 symbols left before WBC + SS to 47.8 ± 6.2 after WBC + SS (*p* = 0.0001) and after 2nd minute decreased from 12.7 ± 11.5 symbols left before WBC + SS to 6.1 ± 7.9 after WBC + SS (*p* = 0.0001). In control group number of symbols left after 1st minute decreased from 55.2 ± 7.3 symbols left before WBC + SS to 49.3 ± 8.1 after WBC + SS (F = 37.4, *p* = 0.0009) and after 2nd minute decreased from 15.1 ± 11.1 symbols left before WBC + SS to 8.1 ± 9.1 after WBC + SS (*p* = 0.001). Results after I and II minute after WBC + SS in CFS group were better than in control group before WBC + SS (47.8 ± 6.2 vs 55.2 ± 7.3 symbols left after I minute, *p* = 0.009 and 6.1 ± 7.9 symbols left after II minute vs 15.1 ± 11.1, *p* = 0.01) (Fig. 5C, D). Data on effects of WBC + SS on cognitive function in CFS and HC groups is summarized in Table S6.

## Discussion

### Effects of WBC

Intensive cooling of the skin surface associated with application of a cryogenic factor at temperatures of − 120 °C causes a number of physiological changes within the cardiovascular and autonomic systems. These effects are mainly by the translocation of blood from within the skin to blood vessels located centrally. Our study has confirmed that WBC is well tolerated by those with CFS and leads to symptomatic improvements associated with changes in cardiovascular and autonomic function. Our results represent preliminary data that will inform discussion as to the physiological impact of low temperatures in those with CFS. Further work is needed.

### Influence of WBC on cardiovascular system and autonomic nervous system function

As an acute response to WBC session skin temperature might diminish because cutaneous microcirculation decrease. This reaction is governed by sympathetic nervous system leading to superficial veno- and vasoconstriction which leads to decrease in blood perfusion through the skin, and increasing central blood flow through large vessels. Higher central blood flow might in consequence lead to higher ventricular filling and the flow of peripheral blood characterized by decreased temperature into the area of the atrial sinus node would impact on heart rate decrease [13]. Centralization of circulating blood after WBC causes a greater filling of large vessels and, consequently, increased ventricular filling, this is confirmed by a significant change in cardioimpedance parameters, i.e. an increase in left ventricular ejection and stroke index. In summary, acute response on WBC session seems to involve modulation of HR and SV what impact on the subsequent physiological reactions [16–18].

### Effects of WBC on fatigue and daytime sleepiness

In the current study, fatigue and daytime sleepiness decreased in the CFS group in response to WBC. To the Authors best knowledge, this is the first study that explores the application of WBC therapy in CFS patients. A WBC programme similar to that applied in the current study was shown to improve health-related quality of life in fibromyalgia patients [22]. The benefits of WBC in fatigue-related symptom reduction have been observed previously in groups of healthy subjects. WBC led to a decrease in the perception of muscular tiredness and pain after physical exercise compared to other recovery interventions [23]. A WBC programme in elite female synchronized swimmers, applied following training sessions, improved sleep quantity during the period of intensive training programme before the Olympic Games [24]. Schaal et al. suggested that the sleep enhancing effects were related to an increase in post-exercise parasympathetic reactivation [24]. Moreover, reduction of daytime sleepiness in CFS in the current study could be related to a decrease in core temperature after the WBC session that occurs 50–60 min after exposure [18]. A relationship between the circadian rhythms of core temperature and sleep has been observed [25]. In CFS patients abnormalities in circadian rhythm has also been observed [26]. Therefore, we suggest that future study should further explore the effects of WBC exposure at different times of day upon changes in core temperature, circadian rhythm and associated sleep efficacy improvement in CFS patients.

### Influence of WBC on cognitive function

While the potential benefits of cryotherapy have been relatively well explored in a number of contexts (e.g. sport benefits, muscle damage, inflammation or sleep problems), to our knowledge, this is one of very few studies assessing the impact of short-term exposure to extreme cold on cognitive function, and the first to include patients with CFS [14, 27]. Interpreting our results in a wider context is therefore limited, with the low number of previous studies compounded by their use of different treatment protocols, cognitive tests and the choice of populations under assessment. Within these limitations however, ours and previous studies consistently show a beneficial impact of cryotherapy on cognitive function. Specifically, a recent investigation, using WBC in an older population with mild cognitive impairment, showed an improvement in immediate recall on DemTect test and orientation on Test Your Memory task [28], while three studies in healthy populations, albeit using partial extreme cold application to the head [29], cheeks [30], and the body apart from the head [31], showed improvement in executive function, namely in cognitive inhibition measured by the Stroop task [28–31].

The mechanisms related to cognitive improvement after short-term extreme cold application are largely unknown. The only study using fMRI showed an increase in the ventrolateral prefrontal cortex activity in response to cold stimuli, allowing some insight into potential mechanisms at the neural level [30]. Another study, which combined an assessment of cognitive and autonomic functions, proposed that improvement in cognitive performance was related to a greater parasympathetic cardiac control and a greater cerebral O2 extraction following a PBC session [31]. Similar to Theurot et al, we also observed an increase in the parasympathetic function after WBC (seen as a decrease in LF, LF/HR ratio, and HR, and an increase in HF) in healthy individuals. In CFS patients, however, these parameters changed in the opposite direction, indicating an increase in the sympathetic tone in this group after cryotherapy. Given that cognitive performance has been consistently shown to worsen with increasing sympathetic tone, and that in our study cognitive improvement was observed in both healthy and CFS groups, changes in autonomic function appear less likely to be the main mechanism through which cryotherapy leads to improvement in cognition [32, 33]. Before any conclusions can be drawn, our findings need replicating; to our knowledge, no other studies exploring this subject in CFS have been published. It also needs to be considered that other factors may contribute to the observed cognitive change. An important one is the learning effect associated with repeated testing, in particular when a test does not have parallel versions that can be applied pre- and post-treatment, as in the case of TMT used in this study [34, 35]. We attempted to mitigate this effect by applying the same tests to both CFS and control groups. However, as both groups improved, at least a partial learning effect cannot be excluded.

Interestingly, both in our, and previous studies, cryotherapy improved selected cognitive abilities, while others were unaffected. For example, Theurot et al. [31] showed an improvement in the reaction time but not accuracy on the Stroop test, while we showed an improvement in the processing speed, cognitive flexibility and executive control but not in DSST set shifting. A meta-analysis by Pilcher et al. [36] showed importance of parameters such as the duration of exposure to the experimental temperature, or the duration of the task, for the effect on cognitive performance [36]. Although only studies using non-extreme temperatures were included, the results suggest that changes in the protocol may have an impact on treatment outcomes. Different parameters will need to be tried, however, a short-term application of extreme cold, as in our study, seems to be crucial for the beneficial effect. Longer exposures to non-extreme cold, e.g. 2 h in 10 °C air [37] or immersion in cold water [38], were shown to have a detrimental effect on cognitive function [37, 38]. This may be linked to differences in physiological reactions triggered by different temperatures and exposure times, or to the shift of attention away from cognitive tasks caused by the experience of prolonged cold, perceived as stress [39, 40].

An important aspect of cryotherapy is its high tolerability. While good tolerance of cryotherapy has been consistently seen in case of many conditions, our study is the first to show it in CFS, with all included participants completing the study [41]. This is in contrast to some other approaches, for example, an aerobic activity programme, from which the dropout rate was about 50% [42, 43]. If replicated, this will be one of the important advantages of cryotherapy.

Given the preliminary data showing the beneficial effect of cryotherapy on cognitive function, its relative ease of application, good tolerability, and proven safety, therapy with extreme cold appears to be an approach worth attention. Further exploration of cryotherapy as a potential treatment for cognitive dysfunction in CFS is particularly important in light of the lack of effective therapeutic options for these common and often disabling symptoms.

### Role of WBC exposure as stressor/similarities with physical activity

A WBC programme might also be considered as physical exercise programme mimetics due to the fact that it induces a pulsative expression of myokines (IL-6, irisin) [14]. Authors suggest that some of the patterns of ANS modulation due to the WBC programme are similar to the effects of physical exercise training [44]. The acute effects of both stressors, namely physical exercise and cold exposure, are related to an increase in stress hormones and sympathetic activity [45]. The acute effects of WBC are related to reduction of resting heart rate and increase in stroke volume [16], stimulates autonomic nervous parasympathetic activity and increases norepinephrine [18, 46]. However, the ratio of stress hormones produced in response to acute physical exercise in comparison to cold exposure might be different [47]. It has been observed that cold exposure leads to an increase in noradrenaline while physical exercise increases the adrenaline level [48]. In our previous study with an intervention based upon a physical activity programme in CFS patients, circa 51% of dropout rate was noted due to PEM [43]. Therefore, both intensity and type of stressor and subtle differences is in hormonal levels could potentially explain the better tolerability of WBC in comparison to programmes based on physical activity that we have used in previous studies.

### Potential mechanism underlying cooling methods effects in CFS

Both pre-, per- and post cooling methods are applied in sport to improve athletes’ performance. Prolonged, intensive physical exercise sessions in high ambient temperature could lead to hyperthermia. Pre-cooling of subjects with physical exercise-induced hyperthermia has been shown to be beneficial for both aerobic and aerobic and cognitive performance, which are disturbed by hyperthermia [49]. The underlying mechanism of pre-cooling effectiveness in sports performance improvement is not yet fully understood [50].

In physical medicine, a variety of cooling methods can be applied, such as WBC, water immersion partial body cryotherapy, local cooling, etc. It seems, that there are differences in the physiological responses to those methods. For instance, a more pronounced decrease in blood flow and tissue temperature were noticed in response to cold water immersion in comparison to WBC [51] and cold-water immersion led to a greater reduction of skin microcirculation in comparison to partial body cryotherapy [52]. WBC induced a greater stimulation of the ANS than partial-body cold exposure [46]. In a previous study, a relatively small drop (0.3 ± 0.2 °C) in core temperature was noticed 60 min after WBC exposure ((− 110 ± 3 °C) for 3 min and 40 s in the main chamber) [53]. The basis of pre-cooling and per-cooling strategies is to reduce the heat stress of the thermoregulatory system prior to, and during, exercise by increasing the heat storage capacity [54, 55]. Several hypotheses on pre-cooling mechanisms have been proposed [50]. Cooling leads to attenuation of the stress of the metabolic and cardiovascular system [56], changes in temperature could influence on function of enzymes [57] and a decrease in the core temperature might attenuate accumulation of blood lactate and the lactate threshold. improvement [58]. In addition, a decrease in core temperature could be related to a reduction of heart rate during physical exercise [59, 60] and cutaneous circulation that leads to a decrease in cardiac filling [58]. In terms of post-cooling methods, a reduction of inflammatory response of muscle tissue to strenuous exercise has been proposed [50]. Peripheral vasoconstriction of the muscle vasculature induced by cold exposure and decrease in muscle tissue temperature might lead to decrease in permeability of cell, lymph and capillaries, which in turn would reduce the diffusion of fluids towards interstitial space and reduce the edema of muscle fiber [61, 62]. The changes in fluids dynamics described above might coexist with attenuated acute inflammation in response to damage of muscle tissue [50]. An increase in metabolic stress might lead to improvement in the mitochondrial energy production, which plays an important role in the production of Reactive Oxygen Species (ROS) in a muscle cell [63]. Therefore, post-cooling might lead to a decrease in ROS production induced by muscle damage, and therefore to reduction in the mitochondrial energy production [50]. In addition, a negative correlation between muscle tissue temperature and nerve conduction velocity have been observed [64, 65]. Both sensory and motor nerve conduction velocity could be reduced [64] which might lead to diminished pain sensation and increase in the pain tolerance [65].

In addition, ATP is maintained at a similar level for at least a day between 10 to 37 degrees of Celsius, however, both utilization and synthesis are negatively corelated with temperature below 37 degrees of Celsius [66].

### Study limitations

Further studies should examine the effects of combined therapies in the management of symptoms in ME/CFS. As Senczyszyn et al. have noted, combinations of Computerized Cognitive Training (CCT), psychoeducation and WBC might be more effective in the reduction of depressive symptoms than Computerized Cognitive Training (CCT) and psychoeducation only [67]. In addition, further studies should consider an RCT design with bigger sample sizes included, with a few months follow-up to examine the sustainability of response. Moreover, a few different WBC protocols should be examined in terms of session’s duration, temperature, frequency and rehabilitation programme duration. In addition, exploring the mechanism by which WBC might decrease fatigue might lead to development of cooling methods that would be more approachable by CFS patients.

We have noted a decrease in daytime sleepiness, however sleep efficacy was not measured objectively in this study. Therefore, the above study results should be replicated on larger sample size with applied long-term follow-up, before recommending WBC as a supportive therapy in CFS.

## Methods

### Setting

This study took place from January 2018 to July 2018 and was approved by the Ethics Committee, Ludwik Rydygier Memorial Collegium Medicum in Bydgoszcz, Nicolaus Copernicus University, Toruń (KB 660/2017). Written informed consent was obtained from all participants.

### Patients

A total of 250 individuals who identified themselves as fatigued, were recruited to the initial evaluation. Individuals who fulfilled the Fukuda criteria for CFS were eligible to enter the study if they were (1) age between 25 and 65 years, (2) fatigue more than 6 months, were severely fatigued, operationalized as scoring more that 36 on the Fatigue Severity Scale, (3) had at least four of additional symptoms: malaise after exertion, headache, impaired memory and/or concentration, unrefreshing sleep, sore throat, tender lymph nodes (cervical or axillary), muscle or joint pain, (4) the fatigue must not be the result of an organic disease [68]. Patients could participate in this study if they had been referred by a general practitioner, neurology and psychiatry; pre-test health state assessment of subjects included: basic neurological, psychiatric, physical examination. The consultant (neurologists or psychiatrists experienced in ME/CFS) confirmed the inclusion and exclusion criteria and verified whether an extensive physical examination and laboratory research tests had been performed to exclude any underlying illness. Where an underlying illness was suspected, the patient was referred to a specialist in internal medicine, neurology or psychiatry for further investigation. An interview with a psychologist was scheduled if the HADS depression subscale score was 11 or more (to exclude a major or bipolar depressive disorder) or if the consultant suspected another psychiatric illness or motivational problem.

Sample size calculation was made using GLIMMPSE 3.0.0 on-line available calculator for General Linear Mixed Model Power and Sample Size (available at: https://glimmpse.samplesizeshop.org/#). Power was set on 0.9, alpha was set on 0.05. Hotelling Lawley Trace test was chosen, as it was advised to choose that option because of similarity with mixed effect linear model. Main effect of WBC + SS on score in FIS was chosen as the primary outcome of the study and decrease from 93.59 to 61.68 and standard deviation of 24.86 points in the CFS group was assumed based on our observation in a previous non-pharmacological intervention based study on CFS patients [69]. Two means scale factors were taken into account: 1 and 0.9. Total sample size was calculated from 37 to 45 participants, respectively (decimal places were rounded). Enrolment ratio was set on 1:0.65 for CFS to control group, because of predicted higher dropout rate in the CFS group. Assuming dropout rate of at least 11%, it was decided to enroll 50 participants in total into study. To obtain 32 CFS patients, 250 patients were initially assessed for eligibility. One hundred and eighty subjects were excluded as they did not meet the Fukuda criteria (n = 180), had an underlying psychiatric illness (n = 30), had another diagnosis or fatigue was not the primary complaint (n = 8). Eighteen healthy individuals were also recruited from the community as a control group (HC).

### Intervention—whole body cryotherapy (WBC)

This was an experimental design with control group. The WBC procedures were performed in a liquid nitrogen cryochamber which consists of two compartments: the antechamber and the proper chamber, which were connected by a door. In the trial, the temperature in the antechamber was − 60 °C, whereas in the proper chamber, it reached − 120 °C.

**Fig. 6:**
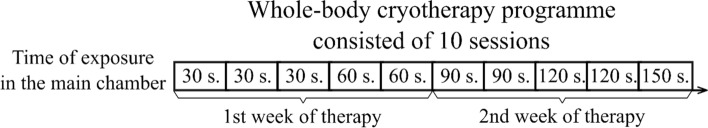
Whole-body cryotherapy programme

The participants were treated using a cycle of 10 visits to a cryogenic chamber over a period of 12 days (from Monday to Friday, scheduled at the same time of day on 5 p.m., one session per day). They were subjected to the effects of extreme low temperature in a WBC chamber for 0.5 to 2.5 min depending upon of the day of therapy, exposure time was incremental (1–3 day for 0.5 min, 4–5 day for 1 min, 6–7 day for 1.5 min, 8–9 day for 2 min, 10 day for 2.5 min), Fig. 6.

In order to protect the most sensitive body areas, all patients entering the rooms were dressed with swimsuits, face-mask to protect the nose and mouth, cotton socks, slippers and gloves, ear-protector and wooden shoes. All jewellery, glasses, and contact lenses were removed before entry into the chamber. During the WBC procedure, individuals walked round the chamber without touching each other.

Immediately after leaving the cryogenic chamber and changing into track suits and trainers, patients underwent kinesiotherapy lasting 30 min. Kinesiotherapy procedures included breathing exercises and passive stretching exercises of the muscles of the major joints (including the ankle, knee, hip, wrist, elbow, shoulder, thoracolumbar spine, and cervical spine). Exercises were introduced in the order from superior to inferior parts of body. Each stretch was held to the point of slight discomfort (not pain) for a period of 20 s per muscle group followed by a 10 s passive rest period in a neutral position [70]. Each stretch was executed once for each limb. Then, during period of rest, supervisor was presenting a following stretch. All the exercises were carried out under the supervision of experienced physiotherapists.

### Measures

The clinical examination was performed in the chronobiology laboratory (windowless and sound-insulated room, temperature 22 °C, humidity 60%) at approximately the same time of day.

#### Fatigue measurements

Fatigue severity was evaluated using three validated tools commonly used to evaluate physical and mental fatigue in patients with CFS; the Chalder Fatigue Questionnaire (CFQ), Fatigue Impact Scale (FIS) and Fatigue Severity Scale (FSS).

The Chalder Fatigue Questionnaire consists of 11 items separated into two dimensions of fatigue severity—mental fatigue and physical fatigue. Seven items represent physical fatigue (items 1–7) and 4 represent mental fatigue (items 8–11). This scale was scored in “Likert” style asking individuals 0, 1, 2 & 3 with a range from 0 to 33 [71].

The Fatigue Impact Scale (FIS) assesses the impact that subjective fatigue has on daily functioning. Forty items are each scored on a 5-point Likert scale (0–4) providing a continuous scale of 0–160 with a higher score indicating greater impact [72].

The Fatigue Severity Scale (FSS) is a short questionnaire that rates the level of fatigue. The FSS questionnaire contains nine statements that rate the severity of your fatigue symptoms on a scale of 1 to 7, based on how accurately it reflects the condition during the past week. A low value (e.g., 1) indicates strong disagreement with the statement, whereas a high value (e.g., 7) indicates strong agreement. A total score of less than 36 suggests no fatigue. A total score of 36 or more suggests fatigue [73].

#### Assessment of daytime sleepiness

Epworth sleepiness scale (ESS) [74] was used to assess patients’ general daytime sleepiness. Higher score indicates higher sleepiness.

#### Assessment of cognitive function

Trail Making Test is a neuropsychological tool offering a rapid assessment measuring various skills including executive functioning domain: visuospatial skills, task switching and working memory [75]. Trial Making Test part A (TMT A) requires to connect dots with numbers from 1 to 25 in sequential order. The task in Trial Making Test part B (TMT B) is to alternate connecting points, connecting the number with the letter, and then again with the number in ascending order (1-A-2-B, etc.). TMT B includes number from 1 to 13 and letters from A to L. Results of TMT A and B were the time to complete the task measured in seconds. Result of TMT A is an indicator of visual search and motor speed skills [76], whereas part B is considered also to be a test of cognitive flexibility and executive control [77–79].

Digit symbol substitution test (DSST) includes digit-symbol pairs and list of digits. Subjects’ task was to write down corresponding symbol to every digit in a consecutive manner as fast as possible [79]. Results were the number of digits left to be encoded after 60 and 120 s. If subject was not able to finish the task within 120 s, the task has been interrupted. DSST result is an indicator of motor speed, attention, scanning, associative learning, executive functions and working memory [80].

#### Autonomic and cardiovascular symptoms measurements

##### Subjective assessment of autonomic symptoms

Participants completed the Autonomic Symptom Profile [81] as a self-report measure of autonomic symptoms. Scoring was performed using the recently abbreviated and psychometrically improved version of this questionnaire, the Composite Autonomic Symptom Score 31 (COMPASS 31) [82]. In addition, Orthostatic Grading scale (OGS) was used to asses response on orthostatic stress [83].

##### Objective assessment of autonomic and cardiovascular function

Cardiovascular and autonomic measurements were performed with a dedicated high-tech device—Task Force Monitor (TFM, CNSystems, Medizintechnik, Graz, Austria). The main area of TFM application is as an automated and computed beat-to-beat analysis of heart rate (electrocardiogram (ECG)) oscillometric and non-invasive continuous blood pressure measurements (oscBP, contBP) [84].

The hemodynamic and left ventricular function parameters analysed were heart rate [HR], systolic blood pressure [sBP], diastolic blood pressure [dBP], mean blood pressure [mBP], stroke index [SI], cardiac index [CI], total peripheral resistance index [TPRI], index of contractility [IC], acceleration index [ACI], left ventricual ejection time [LVET], left ventricular work index [LVWI], total fluid content [TFC], Heather index [HI], total artery compliance [TAC]; autonomic parameters: low frequency (LF), high frequency (HF) of heart rate variability (HRV) and blood pressure variability (BPV), ratio of LF to HF [LF/HF], baroreceptor slope mean. TFM automatically provides a power spectral analysis for HRV BPV which is conducted using the adaptive autoregressive model. All parameters were automatically measured at rest (15 min after stabilization of the signals) and during an active standing test (AS).

### Statistical analysis

All data are presented as mean ± SD. Applicability of parametric tests were verified with the Shapiro–Wilk test and by visual inspection of histograms. To test the effects of WBC (before vs after) in CFS group on variables which values did not meet the normality of distribution assumption, were analysed using Wilcoxon signed-rank test, otherwise paired t-test has been used. To analyse the effects of WBC (before vs after) in groups (HC vs CFS), a linear model with random effect was applied using R statistical packages (Lme4 and LmerTest) were used [85] with a fixed within-subject, fixed between-subject and interaction between those factors. Subject factor was considered as a random. Within-subject factor was effect of WBC + SS (before vs after) and the between-subject factor was group (CFS vs HC), while subject was chosen as a random effect. Package car was used to conduct type III ANOVA on the linear mixed models to test significance of group*effects WBC + SS interaction [86]. Models were fitted using REML and *p*-values for the main effects and their interaction are derived using the Satterthwaite approximation [87]. Results of both effects of time, group and interaction between time and group (time * group) are reported. For significant main effect of WBC + SS, and interaction between effects of group * WBC + SS both F and *p*-values are provided and post-hoc tests analysis was conducted. Result of post-hoc tests were adjusted using Holm correction using lsmeans and multcomp packages [88].

## Supplementary Information

**Additional file 1: Table S1.** Basic characteristics of the CFS/ME group and HC group. **Table S2.** Clinical characteristics of the CFS/ME group. **Table S3.** Details of presenting complaints of CFS/ME group. **Table S4.** Mean ± SD resting values of cardiovascular function indicators for subjects with CFS and HC. **Table S5.** Mean ± SD resting values of autonomic measures for subjects with CFS and HC. **Table S6.** CFS group mean values ± SD before-, after, and follow-up WBC + SS intervention for cognitive function

## Data Availability

The datasets generated during and/or analysed during the current study are available from the corresponding author on reasonable request.
