# Supplementary material for: Combination of whole body cryotherapy with static stretching exercises reduces fatigue and improves functioning of the autonomic nervous system in Chronic Fatigue Syndrome
Source: J Transl Med. 2022 Jun 17;20:273. doi: 10.1186/s12967-022-03460-1 (PMC9204866; doi:10.1186/s12967-022-03460-1)
Supplement: Supplementary file 1 — Additional file 1: Table S1. Basic characteristics of the CFS/ME group and HC group. Table S2. Clinical characteristics of the CFS/ME group. Table S3. Details of presenting complaints of CFS/ME group. Table S4. Mean ± SD resting values of cardiovascular function indicators for subjects with CFS and HC. Table S5. Mean ± SD resting values of autonomic measures for subjects with CFS and HC. Table S6. CFS group mean values ± SD before-, after, and follow-up WBC + SS intervention for cognitive function [file 12967_2022_3460_MOESM1_ESM.docx]

**Supplementary Information**

Table S1. Table Basic characteristics of the CFS/ME group and HC group

| Basic characteristic | CFS/ME group | HC group | p-value |
| --- | --- | --- | --- |
| Number, n | 32 | 18 |  |
| Age, years [Mean±SD] | 36.7±8.4 | 38.4±7.9 | 0.495 |
| Gender, n [F/M] | 26/6 | 10/8 | 0.094 |
| Alcohol use [g] | 15.2±3.6 | 38.3±32.5 | 0.025 |
| Current smoker, n [%] | 0 (%) | 5 (0.9%) | <0.05 |
| Body height, cm [Mean±SD) | 170.4±8.3 | 172.6±9.8 | 0.413 |
| Body mass, kg, [Mean±SD] | 72.2±12.7 | 77.6±20.3 | 0.247 |
| BMI, kg/m^2^, [Mean±SD] | 24.8±3.6 | 25.8±5.7 | 0.395 |
| Body composition |  |  |  |
| BMR [kcal] | 6477.5±1010.1 | 7029.1±1624.2 | 0.145 |
| FatP [%] | 27.7±7.5 | 26.4±7.8 | 0.570 |
| FatM [%] | 20.4±8.0 | 21.2±10.5 | 0.751 |
| FFM [kg] | 51.8±8.7 | 56.4±12.9 | 0.137 |
| TBW [kg] | 37.1±6.1 | 40.2±9.0 | 0.150 |
| PMM [kg] | 49.2±8.3 | 53.6±12.3 | 0.136 |
| VFatL | 4.6±2.4 | 5.9±4.1 | 0.175 |
| BoneM [kg] | 2.6±0.4 | 2.8±0.6 | 0.156 |
| ECW [kg] | 15.8±2.4 | 16.9±3.5 | 0.206 |
| ICW [kg] | 21.2±3.9 | 23.3±5.6 | 0.136 |
| WaterM [%] | 36.4±7.1 | 40.2±9.0 | 0.110 |
| WaterP [%] | 50.8±7.2 | 52.5±5.6 | 0.405 |
| Profession [n] |  |  |  |
| physical work | 0 | 0 |  |
| specialist | 27 | 11 |  |
| management | 2 | 1 |  |
| technicians | 1 | 1 |  |
| off-work / student | 1 | 0 |  |

Table S2. Clinical characteristics of the CFS/ME group

| **Clinical characteristic** | **CFS/ME group** |
| --- | --- |
| Number, n | 32 |
| Length of history | 3.6±2.9 |
| 6 months - 2 years , n [%] | 17 (53.1%) |
| 3 years - 5 years, n [%] | 10 (31.2%) |
| 6 years – 10 years, n [%] | 4 (12.5%) |
| >10 years , n [%] | 1 (3.1%) |
| Used medications |  |
| Contraceptions | 2 (6.2%) |
| Slight antiallergics | 1 (3.1%) |
| Hormones | 22 (68.7%) |
| Slight antidepresants | 1 (3.1%) |
| Dietary supplements | 13 (40.6%) |
| Painkillers | 3 (9.4%) |
| Beta-blockers | 0 (0%) |
| Other | 13 (40.6%) |
| Past medical history, n [%] |  |
| Ischaemic heart disease | 0 (0%) |
| Cerebrovascular disease | 0 (0%) |
| Arthritis | 10 (31.2%) |
| Cardiac arrhythmia | 0 (0%) |
| Depression | 3 (9.4%) |
| IBS | 7 (21.9%) |
| Renal disease | 2 (6.2%) |
| Epilepsy | 0 (0%) |
| Hypertension | 0 (0%) |
| Cognitive impairment | 9 (28.1%) |
| Coeliac disease | 0 (0%) |
| Visual problems | 3 (9.4%) |
| Vertigo | 13 (40.6%) |
| Fibromyalgia | 10 (31.2%) |
| Migraine | 9 (28.1%) |
| Diabetes Mellitus | 1 (3.1%) |
| Gastrointestinal problems | 3 (9.4%) |

Table S3. Details of presenting complaints of CFS/ME group

| **Details of presenting complaints** | **CFS/ME group** |
| --- | --- |
|  |  |
| Number, n | 32 |
| Post exertional fatigue | 23 (71.9%) |
| Long recovery period from exertion | 11 (34.4%) |
| Fatigue | 28 (87.5%) |
| Sleep disturbance | 25 (78.1%) |
| Muscle Pain | 28 (87.5%) |
| Memory disturbance | 27 (84.4%) |
| Confusion and difficulty concentrating | 28 (87.5%) |
| Difficulty retrieving words or saying the wrong word | 28 (87.5%) |
| Gastrointestinal disturbance (diarrhea, IBS) | 18 (56.2%) |
| Recurrent sore throat | 16 (50%) |
| Recurrent flu-like symptoms | 20 (62.5%) |
| Dizziness or weakness upon standing | 21 (65.6%) |
| Change in body temperature. erratic body temperature. cold hand and feet | 25 (78.1%) |
| Heat/cool intolerance | 13 (40.6%) |
| Hot flushes. sweating episodes | 17 (59.4%) |
| Marked weight change | 13 (40.6%) |
| Breathless with exertion | 18 (56.2%) |
| Tender lymph nodes | 9 (28.1%) |
| Muscles weakness | 17 (53.1%) |
| New sensitivities to food / medications /chemicals | 8 (25%) |
| Irritability | 21 (65.6%) |
| Skin changes | 10 (31.2%) |
| Dry eyes. mouth. nose | 19 (59.4%) |

Table S4. Mean±SD resting values of cardiovascular function indicators for subjects with CFS and HC

| **Parameter [unit]** | **CFS group, n=32** | | **HC group, n=18** | | **p-value effects of WBC+SS** | **p-value group** | **p-value WBC+SS*group** |
| --- | --- | --- | --- | --- | --- | --- | --- |
|  | **Before** | **After** | **Before** | **After** |  |  |  |
| **HR [n/1]** | 70.0±1.7 | 72.3±1.7 | 75.6±2.2 | 72.1±2.3 | 0.05 | 0.95 | 0.004 |
| **sBP [mmHg]** | 116.6±1.9 | 111.5±2.4 | 119.0±2.5 | 113.6±3.2 | 0.01 | 0.55 | 0.95 |
| **dBP [mmHg]** | 79.2±1.7 | 75.9±2 | 80.3±2.3 | 76.5±2.6 | 0.03 | 0.84 | 0.86 |
| **mBP [mmHg]** | 95.8±1.7 | 91.3±2.1 | 96.4±2.3 | 91.8±2.9 | 0.01 | 0.87 | 0.97 |
| **SI [ml/m^2^]** | 54.2±2.1 | 50.3±2.2 | 49.0±2.8 | 46.2±2.9 | 0.01 | 0.25 | 0.61 |
| **CI [l/min/m^2^]** | 3.8±0.2 | 3.6±0.2 | 3.7±0.2 | 3.3±0.2 | 0.16 | 0.27 | 0.25 |
| **TPRI [dyne*s*m2/cm5]** | 2127.6±112.6 | 2185.0±143.5 | 2168.0±150.1 | 2351±191.3 | 0.51 | 0.44 | 0.38 |
| **IC [1000/s]** | 66.1±21.5 | 60.0±21.5 | 58.4±19.2 | 52.6±18.2 | 0.01 | 0.23 | 0.94 |
| **LVWI [mmHg*l/[min*m^2^]]** | 4.8±0.2 | 4.2±0.2 | 4.7±0.3 | 3.9±0.2 | 0.0001 | 0.32 | 0.33 |
| **LVET [ms]** | 316.8±2.4 | 313.6±2.7 | 305.4±3.2 | 307.3±3.6 | 0.11 | 0.15 | 0.13 |
| **TFC [1/Ohm]** | 32.2±1.0 | 33.3±1.0 | 33.9±1.3 | 32.3±1.4 | 0.06 | 0.56 | 0.01 |
| **ER [%]** | 36.73±3.4 | 37.21±3.4 | 38.15±3.9 | 36.51±3.9 | 0.25 | 0.51 | 0.003 |
| **MSER [ml/s]** | 311.56±69 | 290.92±70.6 | 301.5±58.6 | 279.1±58.5 | 0.01 | 0.55 | 0.88 |

HR, heart rate; sBP, systolic blood pressure; dBP, diastolic blood pressure; mBP mean blood pressure; SI, stroke index; CI, cardiac index; TPRI , total peripheral index, TAC , total artery compliance IC, index cardiac; LVWI, left ventricular work index; LVET, left ventricular ejection time; HI, Heather index; TFC, thoracic fluid content; ER, ejection rate; MSER, Mean Systolic Ejection Rate. *P*-values from two-way ANOVA conducted on linear mixed models are reported for the effects of group (HC vs CFS), WBC+SS (before vs after) and interaction between those factors

**Table S5.** Mean±SD resting values of autonomic measures for subjects with CFS and HC

| **Parameter [unit]** | **CFS group, n=32** | | **HC group, n=18** | | **p-value effects of WBC+SS** | **p-value group** | **p-value WBC+SS*group** |
| --- | --- | --- | --- | --- | --- | --- | --- |
|  | **Before** | **After** | **Before** | **After** |  |  |  |
| **LFnu-RRI [%]** | 54.5±2.6 | 59±2.7 | 70.3±3.5 | 61.6±3.6 | 0.07 | 0.57 | 0.002 |
| **HFnu-RRI [%]** | 45.5±2.6 | 41±2.7 | 29.7±3.5 | 38.4±3.6 | 0.07 | 0.57 | 0.002 |
| **LF/HF-RRI [n/1]** | 1.9±0.4 | 2.2±0.4 | 3.0±0.6 | 2.1±0.5 | 0.32 | 0.87 | 0.03 |
| **LF/HF [n/1]** | 1.6±0.3 | 2.0±0.3 | 2.5±0.4 | 1.9±0.4 | 0.16 | 0.95 | 0.03 |
| **LFnu-dBP [%]** | 50.9±2.7 | 56.8±3.6 | 59.6±3.6 | 55.7±4.8 | 0.10 | 0.84 | 0.10 |
| **HFnu-dBP [%]** | 13.3±1.6 | 11.1±2.1 | 8.6±2.1 | 12.7±2.8 | 0.31 | 0.61 | 0.09 |
| **LF/HF-dBP [n/1]** | 6.2±1.1 | 9.5±1.3 | 11.4±1.4 | 8.6±1.8 | 0.01 | 0.65 | 0.004 |
| **LFnu-sBP [%]** | 41.4±2.6 | 46.0±2.8 | 52.2±3.5 | 47.9±3.8 | 0.09 | 0.68 | 0.046 |
| **HFnu-sBP [%]** | 15.3±1.6 | 14.9±2.2 | 10.3±2.1 | 17.6±3 | 0.87 | 0.42 | 0.047 |
| **LF/HF-sBP [n/1]** | 3.8±0.6 | 4.7±0.6 | 6.8±0.8 | 4.5±0.8 | 0.17 | 0.85 | 0.003 |

LF-RRI, low frequency R-R interval; HF-RRI, high-frequency R-R interval, PSD-RRI, power spectral density R-R interval; LF/HF, ratio between low and high band for heart rate and blood pressure variability; PSD-sBP, power spectral density of systolic blood pressure variability; LF-sBP, low frequency of systolic blood pressure variability; HF-sBP, high frequency of systolic blood pressure variability; PSD-dBP, power spectral density of diastolic blood pressure variability; LF-dBP, low frequency of diastolic blood pressure variability; HF-dBP, high frequency of diastolic blood pressure variability. *P*-values from two-way ANOVA conducted on linear mixed models are reported for the effects of group (HC vs CFS), WBC+SS (before vs after) and interaction between those factors

**Table S6***.* CFS group mean values±SD before-, after, and follow-up WBC+SS intervention for cognitive function

| **Parameter [unit]** | **CFS group, n=32** | | **HC group, n=18** | | **p-value effects of WBC+SS** | **p-value group** | **p-value WBC+SS*group** |
| --- | --- | --- | --- | --- | --- | --- | --- |
|  | **Before** | **After** | **Before** | **After** |  |  |  |
| **TMT A [s]** | 23±6.2 | 18.4±5.7 | 24.5±7.6 | 20.9±8.9 | 0.000003 | 0.23 | 0.36 |
| **TMT B [s]** | 50.2±13.9 | 43.3±11.3 | 59.7±25 | 50±23.7 | 0.005 | 0.21 | 0.61 |
| **TMT B-A [s]** | 27.2±11.4 | 25±9.5 | 35.2±23.7 | 29.1±21 | 0.31 | 0.38 | 0.36 |
| **Coding 1 min [symbols_to_go]** | 52.8±9.5 | 47.8±6.2 | 55.2±7.3 | 49.3±8.1 | 0.00002 | 0.52 | 0.70 |
| **Coding 2 min [symbols_to_go]** | 12.7±11.5 | 6.1±7.9 | 15.1±11.1 | 8.1±9.1 | 0.00001 | 0.49 | 0.91 |

TMT A (Trial Making Test part A); TMT B (Trial Making Test part B); Coding 1 min (number of symbols left to go after 1 minute in Coding); Coding 2 min (number of symbols left to go after 2 minutes in Coding). *P*-values from ANOVA conducted on linear mixed models are reported for the effects of group (HC vs CFS), WBC+SS (before vs after) and interaction between those factors
